# Screening and Early Diagnosis in Gynecological Cancers

**DOI:** 10.3390/cancers15215152

**Published:** 2023-10-26

**Authors:** Luca Giannella, Andrea Ciavattini

**Affiliations:** Woman’s Health Sciences Department, Gynecologic Section, Polytechnic University of Marche, 60123 Ancona, Italy

Cervical (CC), endometrial (EC), and ovarian (OC) cancers are the pathologies with the highest incidences among gynecological tumors, with such high morbidity and mortality values that they are considered significant public health problems. In this regard, various screening or early detection types have been tested for these diseases. A comprehensive and fascinating review on this topic was reported by Holcakova et al., focusing on the role of liquid-based biomarkers as a screening or early cancer detection option in this area [1]. Unlike traditional biopsies, liquid biopsies allow us to study the presence of tumor material in human fluids, helping diagnose tumors with poor anatomical accessibility [2,3]. Given the disappointing endometrial and ovarian cancer results, this review offers interesting insights for future research directions. As reported, liquid biopsies include circulating tumor cells (CTC), circulating tumor DNA (ctDNA), circulating cell-free RNA, and circulating extracellular vesicles called exosomes [1,4].

## 1. Cervical Cancer Screening

CC is the only gynecological cancer for which there is primary and secondary prevention. Initially, the only screening test for cervical cancer was the Pap test. As previously reported, the limitations of the Pap test lie in its low sensitivity. A meta-analysis showed a pooled sensitivity value of 61.1% in detecting CIN2+ lesions [5]. In 2002, with the introduction of the HPV-DNA test as a cervical cancer screening tool, the sensitivity increased to 94.1% in detecting CIN2+ lesions [5].

CC is also the only cancer that has a vaccine as a prophylactic preventive strategy. In 2006, the FDA approved the bivalent HPV vaccine, including genotypes 16 and 18 [6]. Subsequently, quadrivalent and nonavalent HPV vaccines were approved in 2007 and 2014, respectively [6,7]. The latter includes genotypes 6, 11, 16, 18, 31, 33, 45, 52, and 58, responsible for 90% of CCs and most glandular lesions [8,9]. Despite primary and secondary prevention for CC, it is currently one of the most common gynecological cancers among women, with 80% of cases in developing countries [5]. The World Health Organization has set the primary objective of eliminating CC by 2030, a result achievable with an incidence of fewer than four cases of cervical cancer per 100,000 woman years [10]. For this goal, the WHO has established the following vaccination and screening coverage cut-offs: (a) “90% of girls fully vaccinated with the HPV vaccine by 15 years of age”; and (b) “70% of women screened with an HPV test at 35 and 45 years of age and all managed appropriately” [10]. However, vaccination and screening coverage worldwide is well below these values, representing a significant public health problem [10]. In December 2022, to increase vaccination coverage globally by reducing costs and resources, the WHO recommended a single dose of the HPV vaccine in girls aged 9–14 years as an effective strategy against CC [11].

It is clear from the above that our efforts must mainly aim to increase vaccination and screening coverage in the target population. However, as reported by Holcakova et al., there are novel biomarkers in liquid-based cytology that may represent additional tools for detecting cervical lesions [1]. It has been highlighted that the most promising biomarker is the detection of HPV E6/E7 mRNAs [1]. This methodology showed a sensitivity of 91.6% and a specificity of 98.6% for high-grade cervical lesions [12]. A positive HPV test does not discriminate between infections that will regress or progress. We know that the host will clear many HPV infections. This aspect explains the limited specificity of the HPV DNA test. On the contrary, the detection of HPV RNA E6/E7 is an expression of the oncogenic activity of the virus, probably better identifying those infections with the real risk of transforming action [13]. Their clinical utility, therefore, may include the triage of HPV-positive women and their follow-up [13]. 

A further and investigated epigenetic mechanism is DNA methylation. As highlighted in the review, methylation rates correlate with disease stage in low- and high-grade cervical lesions and invasive cancers [1]. Furthermore, studying HPV DNA methylation in HPV-positive women is an exciting research topic. Increased HPV methylation correlates with a higher risk of severe cervical lesions [1]. These data are relevant from a diagnostic point of view and provide the basis for new therapeutic strategies based on demethylating treatments [14]. However, the methylation test is not included in clinical practice. This missed step is because studies on this topic are very heterogeneous regarding the genes studied and the methodological approach, resulting in study protocols that are very different from each other [14].

These new and promising diagnostic tools aim to increase the specificity of the HPV DNA test, potentially leading to a decrease in overdiagnosis and overtreatment, as well as costs, women’s quality of life, and the number of unnecessary colposcopies.

## 2. Endometrial Cancer Screening

EC represents the most common disease in developed countries. Usually, it shows signs of itself early with postmenopausal vaginal bleeding in 90% of cases and has an average 5-year survival of 80% [15]. To date, there is no screening method for EC. Endometrial sampling via brush or pipelle has been the most studied screening method. However, it provided sensitivity values between 56–100% and 38–100%, depending on whether the histological gold standard was represented by hysterectomy or D&C, respectively [16]. The role of transvaginal ultrasound in measuring endometrial thickness provided sensitivity and specificity values of 80% and 86% in asymptomatic postmenopausal women with endometrial thickness of 5 mm without improving the mortality rate [17]. In women with high risk for EC, such as in the case of autosomal dominant Lynch syndrome, a prophylactic hysterectomy should be performed after completing childbearing. Before definitive treatment, a gynecological evaluation and transvaginal ultrasound should be performed annually, starting from age 25 [18].

In light of these data, the study of new markers could be interesting, as reported in the review [1]. While uterine aspirate using proteomic technology can aid in the diagnosis of EC, the study of CTCs provides information on the prognosis of the disease. cfDNA may have a role in prognosis and via Next Generation Sequencing technology in diagnosis. The same goes for circulating miRNAs. Finally, the study of ctDNA can be helpful to verify the response to treatment [3]. Although their use in screening appears untested, they, alone or in combination, could have a clinical impact on identifying better patients with residual disease or at risk of recurrence and in targeted therapy aimed at improving personalized medicine.

## 3. Ovarian Cancer Screening

OC is the most lethal gynecological malignancy, with a median 5-year survival of 30%. Unfortunately, this type of cancer is diagnosed late in about 2/3 of cases [19]. For this reason, achieving an early diagnosis would represent a result of significant clinical relevance. Two randomized controlled trials using the plasma Ca-125 value and ultrasound evaluation showed no benefits in terms of mortality in women undergoing this type of screening, increasing the harm in women subjected to close monitoring [20,21]. Even in at-risk women carrying the BRCA-1/BRCA-2 mutation, annual Ca-125 and ultrasound evaluations have been reported to have poor sensitivity. The only option that has shown a significant reduction in the risk of developing OC in these women is bilateral salpingo-oophorectomy between the ages of 35–40 or soon after completing childbirth [22].

Given the disappointing results, the review by Holcakova et al. comprehensively provides clinical insights based on liquid biopsies [1]. The authors reported data regarding protein biomarkers, CTCs, Circulating Free and Circulating Tumor DNA, Exosomes, and Circulating Cell-Free MicroRNAs [1]. By summarizing and underlining the practical clinical aspects, these biomarkers could help for an earlier diagnosis, detect residual disease after surgical debulking, and anticipate the diagnosis of recurrence. Furthermore, liquid biopsies capture the entire tumor genome, possibly identifying genetic markers predictive of success or resistance to therapy [23].

## 4. Future Directions

As mentioned, liquid biopsies represent a field of study to improve the diagnosis, follow-up, and treatment of these pathologies. However, another innovative field for cancer screening should also be highlighted. Artificial intelligence (AI) has revealed interesting perspectives regarding diagnosis, prognosis, and CC, EC, and OC treatment. AI showed prediction values superior to human assessment using data from medical records, radiological imaging, liquid cytology, colposcopic images, and molecular profiling [24]. In this regard, AI could be combined with liquid biopsies to further improve the diagnostic–therapeutic work-up of these diseases in clinical practice. However, it must be emphasized that there has yet to be validation data on large populations.

## 5. Screening and Early Diagnosis of Disease

Although the topics mentioned above represent the future of research in this area, we still need to be able to apply these methods in a real screening setting. It should not be forgotten that an organized cancer screening program is a complex system, including leadership, governance, finance, health workforce, access to essential services, service delivery provisions, information systems, and quality assurance.

Screening and early cancer detection are different concepts, but both are strategies for early disease diagnosis. Screening is aimed at asymptomatic women at risk of the disease, whereas early diagnosis involves symptomatic women at an early stage. While screening involves testing many patients, uses many resources, and is a very complex system, in contrast, early cancer detection can involve fewer patients, resources, and a much less complex organizational system.

The diagnostic accuracy of screening is measured through sensitivity, specificity, positive predictive value (PPV), and negative predictive value values (NPV). A practical screening test should have a sensitivity more significant than 70% and a specificity greater than 95% [25]. The PPV and NPV depend on the prevalence of the disease and are directly and inversely related to it, respectively.

According to the Wilson and Jugner criteria, the WHO has included the following four criteria for a disease to be suitable for screening [26]:

- “The condition should be a major health problem with a latency or early detection phase”;
- “The test should be simple, safe, precise, validated and acceptable to the population”;
- “There should be evidence that early treatment improves outcomes compared to late treatment”;
- “The screening program should have evidence from high-quality randomized controlled trials that it is effective in reducing mortality or morbidity and that the benefits of screening should outweigh the physical and psychological harms”.

Finally, lead time bias must also be considered when evaluating a screening test. It represents a phenomenon whereby cancer is detected earlier, but the time to death does not change. It provides an increased impression of survival without prolonging the patient’s life. This phenomenon can make screening much more effective than it is, and this aspect must be included in the risk/cost/benefit ratio of screening [25].

## References

[1] Holcakova J., Bartosik M., Anton M., Minar L., Hausnerova J., Bednarikova M., Weinberger V., Hrstka R. (2021). New Trends in the Detection of Gynecological Precancerous Lesions and Early-Stage Cancers. Cancers.

[2] Baev V., Koppers-Lalic D., Costa-Silva B. (2023). Liquid Biopsy: Current Status and Future Perspectives. Cancers.

[3] Muinelo-Romay L., Casas-Arozamena C., Abal M. (2018). Liquid Biopsy in Endometrial Cancer: New Opportunities for Personalized Oncology. Int. J. Mol. Sci..

[4] Armakolas A., Kotsari M., Koskinas J. (2023). Liquid Biopsies, Novel Approaches and Future Directions. Cancers.

[5] Terasawa T., Hosono S., Sasaki S., Hoshi K., Hamashima Y., Katayama T., Hamashima C. (2022). Comparative accuracy of cervical cancer screening strategies in healthy asymptomatic women: A systematic review and network meta-analysis. Sci. Rep..

[6] Ciavattini A., Giannella L., De Vincenzo R., Di Giuseppe J., Papiccio M., Lukic A., Delli Carpini G., Perino A., Frega A., Sopracordevole F. (2020). HPV vaccination: The position paper of the Italian society of colposcopy and cervico-vaginal pathology (SICPCV). Vaccines.

[7] Food and Drug Administration FDA Approves Gardasil 9 for Prevention of Certain Cancers Caused by Five Additional Types of HPV. https://www.esmo.org/oncology-news/archive/fda-approves-gardasil-9-for-prevention-of-certain-cancers-caused-by-five-additional-types-of-hpv.

[8] Giannella L., Giorgi Rossi P., Delli Carpini G., Di Giuseppe J., Bogani G., Gardella B., Monti E., Liverani C.A., Ghelardi A., Insinga S. (2021). Age-related distribution of uncommon HPV genotypes in cervical intraepithelial neoplasia grade 3. Gynecol. Oncol..

[9] Giannella L., Delli Carpini G., Di Giuseppe J., Bogani G., Sopracordevole F., Clemente N., Giorda G., De Vincenzo R.P., Evangelista M.T., Gardella B. (2023). In Situ/Microinvasive Adenocarcinoma of the Uterine Cervix and HPV-Type Impact: Pathologic Features, Treatment Options, and Follow-Up Outcomes-Cervical Adenocarcinoma Study Group (CAS-Group). Cancers.

[10] Cervical Cancer Elimination Iniziative. https://www.who.int/initiatives/cervical-cancer-elimination-initiative#cms.

[11] (2022). Human Papillomavirus VACCINES: WHO Position Paper. https://www.who.int/publications/i/item/who-wer9750-645-672.

[12] Munkhdelger J., Kim G., Wang H.-Y., Lee D., Kim S., Choi Y., Choi E., Park S., Jin H., Park K.H. (2014). Performance of HPV E6/E7 mRNA RT-qPCR for screening and diagnosis of cervical cancer with ThinPrep^®^ Pap test samples. Exp. Mol. Pathol..

[13] Sharma B., Lakhanpal V., Singh K., Oberoi L., Bedi P.K., Devi P. (2022). Evaluation of HPV E6/E7 mRNA Detection in Clinically Suspected Cases of Cervical Cancer with Abnormal Cytology: Time to Upgrade the Screening Protocols. J. Lab. Physicians.

[14] von Knebel Doeberitz M., Prigge E.S. (2019). Role of DNA methylation in HPV associated lesions. Papillomavirus Res..

[15] Yi M., Li T., Niu M., Luo S., Chu Q., Wu K. (2021). Epidemiological trends of women’s cancers from 1990 to 2019 at the global, regional, and national levels: A population-based study. Biomark. Res..

[16] Wang T., Jiang R., Yao Y., Wang Y., Liu W., Qian L., Li J., Weimer J., Huang X. (2023). Endometrial Cytology in Diagnosis of Endometrial Cancer: A Systematic Review and Meta-Analysis of Diagnostic Accuracy. J. Clin. Med..

[17] Jacobs I., Gentry-Maharaj A., Burnell M., Manchanda R., Singh N., Sharma A., Ryan A., Seif M.W., Amso N.N., Turner G. (2011). Sensitivity of transvaginal ultrasound screening for endometrial cancer in postmenopausal women: A case-control study within the UKCTOCS cohort. Lancet Oncol..

[18] Møller P., Seppälä T., Bernstein I., Holinski-Feder E., Sala P., Evans D.G., Lindblom A., Macrae F., Blanco I., Sijmons R. (2017). Cancer incidence and survival in Lynch syndrome patients receiving colonoscopic and gynaecological surveillance: First report from the prospective Lynch syndrome database. Gut.

[19] Sant M., Chirlaque Lopez M.D., Agresti R., Sánchez Pérez M.J., Holleczek B., Bielska-Lasota M., Dimitrova N., Innos K., Katalinic A., Langseth H. (2015). Survival of women with cancers of breast and genital organs in Europe 1999-2007: Results of the EUROCARE-5 study. Eur. J. Cancer.

[20] Jacobs I.J., Menon U., Ryan A., Gentry-Maharaj A., Burnell M., Kalsi J.K., Amso N.N., Apostolidou S., Benjamin E., Cruickshank D. (2016). Ovarian cancer screening and mortality in the UK Collaborative Trial of Ovarian Cancer Screening (UKCTOCS): A randomised controlled trial. Lancet.

[21] Buys S.S., Partridge E., Black A., Johnson C.C., Lamerato L., Isaacs C., Reding D.J., Greenlee R.T., Yokochi L.A., Kessel B. (2011). Effect of screening on ovarian cancer mortality: The Prostate, Lung, Colorectal and Ovarian (PLCO) Cancer Screening Randomized Controlled Trial. JAMA.

[22] Bermejo-Pérez M.J., Márquez-Calderón S., Llanos-Méndez A. (2007). Effectiveness of preventive interventions in BRCA1/2 gene mutation carriers: A systematic review. Int. J. Cancer.

[23] Zhu J.W., Charkhchi P., Akbari M.R. (2022). Potential clinical utility of liquid biopsies in ovarian cancer. Mol. Cancer.

[24] Jiang Y., Wang C., Zhou S. (2023). Artificial Intelligence-based Risk Stratification, Accurate Diagnosis and Treatment Prediction in Gynecologic Oncology. Semin. Cancer Biol..

[25] Marcus P.M. (2022). Cancer Prevention Screening. Assessment of Cancer Screening.

[26] WHO A Short Guide to Cancer Screening: Increase Effectiveness, Maximize Benefits and Minimize Harm. https://iris.who.int/handle/10665/351396?&locale-attribute=pt.

